# Assisted peritoneal dialysis: strategies and outcomes

**DOI:** 10.1186/s41100-021-00390-4

**Published:** 2022-01-10

**Authors:** Anna Giuliani, Luca Sgarabotto, Sabrina Milan Manani, Ilaria Tantillo, Claudio Ronco, Monica Zanella

**Affiliations:** 1 Department of Nephrology Dialysis and Transplantation, San Bortolo Hospital, Vicenza, Italy; 2 International Renal Research Institute Vicenza (IRRIV), Vicenza, Italy

**Keywords:** Assisted peritoneal dialysis, Home-dialysis, Elderly

## Abstract

Assisted peritoneal dialysis (asPD) is a modality intended for not self-sufficient patients, mainly elderly, who are not able to perform peritoneal dialysis (PD) alone and require some help to manage the treatment. In the last decades, many countries developed strategies of asPD to face with aging of dialysis population and give an answer to the increasing demand of health service for elderly. Model of asPD varies according to the type of assistants employed and intensity of assistance provided. Both health care and non-health care assistants have been used with good clinical results. A mixed model of help, using different professional figures for short time or for longer according to patients’ need, has been proved successful and cost-effective. Outcomes of asPD are reported in different ways, and the comparative effect of asPD is unclear. Quality of life has rarely been evaluated; however, patients seem to be satisfied with the assistance provided, since it allows them to both retain independence and to be relieved from the burden of self-care. Assisted PD should not be intended as a PD-favoring strategy, but as a model that allows home dialysis also in patients who would not be eligible for PD because of social, cognitive or physical barriers.

## Background

Assisted PD (asPD) is an increasing model of care in which assistance with peritoneal dialysis (PD) treatment is provided to the patient [1].

In recent years, many countries have implemented strategies of asPD to face the reality of the progressive aging of dialysis population [2]. Data from ERA-EDTA registry Annual Report 2018 showed that over 81,714 individuals starting kidney replacement therapy (KRT) for kidney failure, 51% were over 65 years old, with 25% aged more than 75 years. This means that the large part of our elderly patients is in good clinical condition so that they meet criteria to start a dialysis program. However, the presence of comorbidities and disability make them often unable to perform PD autonomously. Therefore it is not surprising that the percentage of patients who starts PD decreases with increasing age, as it is goes from 24% in patients younger than 19 years, to 12% in those older than 75 years [3].

However, in countries where an asPD program exists, patients on PD are older, compared with those without asPD programs. For example in France, where a well-established asPD program has existed for years, median age at PD start is 71 years [4], compared with an average of 60 years in German [5].

A larger availability of home-care assistance might theoretically increase the amount of patients considered eligible for PD [6]. Although this might ease the start of PD, a long-term effect is less predictable, since prevalence of PD remain low with great variability across the world and even across the same country [7]. Confirming that, even if France has a robust program of asPD, prevalence of PD utilization is low and around 10%, with big regional variability (2.9–26.5%) [8]. The same is true for UK, where a variability between 6.3% and 49.7% is described. This highlights that the low PD prevalence may be due to non-clinical factors [9].

Regardless the reasons, elderly and disabled patients are mostly treated with in-center hemodialysis (HD), despite the fact that if educated and involved in choice, > 50% of them would choose home-based dialysis [10]. Assisted PD opens option of PD in frail patients, which are also the ones who may benefit the most from a home-based treatment [11].

Recently many countries have implemented strategies to support home-based medicine, also thanks to increased tele-health tools [12]. This revolution is involving different fields of medicine, and it started before the Covid-19 outbreak [13]. However, during the last year, we understand even more the importance of being independent form the hospital since we were forced to keep patients home, avoiding unnecessary access to hospital, especially for elderly and frail patients [14, 15].

Nowadays elderly patients account for the most of end stage kidney disease (ESKD) patients. This subset of patients has special needs and requires tailored medicine focused more on quality of life than on survival. Remaining at home has a big impact on quality of life for elderly patients and provision of assistance for the PD procedure in patient’s home, overcomes barriers that otherwise would obstacle its use.

Assisted PD may also be used temporarily in case of unplanned start of PD or during period of respite or in all situations in which patients are unable to continue PD alone.

In this paper, we will review strategies and outcomes of asPD models.

## Main text

### Barriers to peritoneal dialysis

There are many physical and social conditions, which may represent a barrier to PD use in elderly patients. Reduced strength to lift bags, manual dexterity, blindness, deafness, immobility, together with psychological conditions like anxiety, impaired cognition or living alone have been all identified as barriers [16]. Such limitations restrict the use of PD since they make difficult performing both basic and instrumental PD activities.

In a Canadian cohort of 134 incident patients, starting dialysis between 2004 and 2006 with a median age of 73 years, 81% of them had at least one barrier to PD [16]. In an another Canadian cohort including over 121 patients aged 69 + -10 years, starting dialysis between 2012 and 2015, authors found that more than half of patients needed an help in a median of 3 dialysis related tasks, while only 5% were completely independent in PD procedure [17].

To overcome these barriers some patients may be helped from a familial caregiver or can hire a domestic worker, who can be appropriately trained to PD procedures. However, most of the patients cannot find help in the family or cannot afford it. Therefore, many countries have proposed model of asPD publicly funded, mostly based on the employment of health care workers, which may be entirely or only partially involved in PD procedure, according to patient needs [2].

### Models of assisted peritoneal dialysis

Assistance may vary in terms of type of professional figure involved, who may be a health care or not health care professional assistant, and intensity of assistance provided. The choice depends on health care system organizations and reimbursement policy. Assistance may then be used for a limited period or can be offered on long term-basis, depending on the increasing skills with the procedures. In a paper of Oliver et al., it is reported that 23% of assisted PD patients may gradually improve and become autonomous [16]. The same authors reported in a following paper that 38% of asPD patients improved to self-PD or to familial assisted PD within 30 day after dialysis start [18]. Also Bevilacqua et al. described a median time of 29 days to graduate to self-PD for those patients who temporary require assistance [19].

Assisted PD can be performed also in residential homes where trained nurses manage CAPD exchanges or cycler setup and connection/disconnection.

In models where a health care professional assistance is provided, a registered nursed is commonly employed. Alternatively, a nurse assistant or health care aide/worker can be recruited. The main difference between these professional figures is the clinical background (and consequently the higher cost), which allows registered nurse to give a basic judgment regarding fluid assessment, nutrition and blood pressure control and to be more independent and able to give suggestions and advice to the patient.

In France, where a network of private nurses dedicated to home assistance exists, almost 45% of incident PD patients are treated with asPD. Private nurses, who are fully reimbursed by health care insurance, are properly formed by public nephrology department to both CAPD and APD procedures and are supported by an on-call service 24 h a day, 7 days a week from hospital. CAPD assistance requires 3–4 visits per day, even when a non-disconnected system is used, while APD requires maximum twice visits per day, one in the morning to disconnect patient and set up cycler for the next treatment and one in the evening to connect patient to cycler. Number of visits per day can change according to patients learning [20, 21].

During home visits, dialysis nurses regularly check the environment and perform retraining, if necessary.

In Denmark, assistance is provided mainly for APD and is guaranteed by professional publicly paid community nurses or by nursing home staff [21, 22].

UK have a program of asPD started in 2007, based on health care support workers, founded by National Health Service. The program is conceived for APD and assistants may perform one daily visit to set up the cycler, if a familiar caregiver carries out connection and disconnection, or two daily visits if assistant is responsible also for the connections [23].

In 2004, an Italian group in Lombardy also employed publicly paid health workers as assistants. They were trained in dialysis unit to perform both CAPD and APD [24].

In Canada, at the Sunnybrook Health Science in Toronto, asPD started in 2004. At first, they used registered nurses and then adopted a mixed model of assistance, which involved also practical nurses. This model allowed the correct level of care to be assigned according to the patient needs and complexity [16].

In British Columbia, a 12-month pilot program of asPD started in 2015. They selected caregivers without prerequisite level of clinical or health care certification, who completed a PD technique training. These caregivers were responsible for dismantling and setup of the cycler, measuring blood pressures and weight, adding medications to dialysate as prescribed by the PD physicians. Patient or familial caregiver were still responsible for connection/disconnection and troubleshooting during treatment [19] .

In Gamen Clinic in Rio de Janeiro, Brazil, an asPD program employed assistant nurses only for APD [25].

In Italy, Viglino proposed many years ago the idea of a video caregiver to assist patient during PD procedures. Video dialysis includes a remote station at patient’s home and a control station in the hospital, with a software that supports 6 audio–video connections simultaneously [26].

A European survey on asPD practice in Europe has been recently published, showing that being western and Scandinavian countries, non-academic centers and the presence of a dedicated team for education were factors associated with availability of asPD. Scandinavian countries reported a proportion of > 30% for incident and prevalent home dialysis patients [6].

In Honk Kong where a PD-first strategy exists since 1994, PD prevalence reaches 90% and all patients are treated with PD unless contraindicated. They can offer helper-assisted CAPD with three different types of helpers: family members, domestic helpers, and nursing home staff (who are semiskilled health care assistants) [27]. Some other Chinese reports described the use of family-member or domestic worker paid by the employer, as helpers [28], selected according to education, with no health care background required, only able to read and write [29].

Although there are some initiative of asPD worldwide, most of the countries cannot offer a program of assistance for home dialysis yet. In the USA, in July 2019 there was the announcement of Advancing American Kidney health initiative to improve kidney health and demand of kidney transplants and dialysis is reduced. This initiative was also aimed to promote the development of strategies of home-dialysis on medium term basis (2–5 years) [1]. Table 1 summarizes some strategies of asPD.

**Table 1:** Strategies and costs of assistance

Country	Type of asPD provided	Paid caregiver	Costs
France [20]	CAPD/ APD	Private nurse reimbursed directly by the French Healthcare insurance	CAPD: 23,400 € /patient/year
	(up to 4 visits/daily)		APD: 18,200 € /patient/year
Denmark [22]	APD (up to 2 visits daily)	Community nurses or nursing home staff	APD: 54 €/hourly
			16,178 € /patient/year
Canada, Ontario [20]	CAPD/APD (up to 2 visits/day)	Home care nurses	Additional cost of providing assistance 12,000 $ CA (median 4.6 visits/week)
Belgium [11]	CAPD/APD	Registered nurses (reimbursed by patient’s health insurance provider)	CAPD: 9360 € /patient/year (regardless the number of exchanges)
			APD: 5356 € /patient/year (for 2 visits/day)
Spain (Canary island) [11]	CAPD/APD	Nurses	20 €/ day (7280 €/patients/year)
Italy (Milano) [28]	CAPD/APD	Health care workers publicly paid	
United Kingdom [27]	APD (1 visit/daily)	Health care workers	
Canada, British Columbia [23]	APD	Trained caregiver with no prerequisite of clinical or heath care certification	Additional cost of providing assistance 15,000 $ CA
Italy (Alba) [30]	APD/CAPD	Video dialysis	
China [27–29]	CAPD	Family members, domestic workers (paid by the employer), nursing home staff	

AsPD: assisted peritoneal dialysis; CAPD: continuous ambulatory peritoneal dialysis; APD: automated peritoneal dialysis

### Outcomes

Different studies on asPD evaluated several outcomes, such as technique failure, peritonitis risk, hospitalization and mortality. However, only few studies have focused on patient experience and quality of life (QoL), which instead should play a central role given the subset of patients for whom the program is designed. Moreover, even if asPD is considered a PD-favoring program, its effect on PD utilization has rarely been evaluated.

Most of the outcomes analyzed showed great variability in results since patients treated with asPD are typically older and with more comorbidities, than self-PD patients. Conversely, if in-center HD patients are taken as comparison group, bias in selection should be ascertained matching population for baseline characteristics.

Oliver et al. published data about hospitalization rate comparing 203 asPD with 198 in center HD patients. They found similar results in both groups (11.1 vs 12.9 days/year equal to 0.8 vs 0.7 hospital admission per year) with no differences between familial and nurse asPD patients. Assisted PD patients were more likely hospitalized for dialysis related complications compared with HD patients [18].

Similarly, authors of Frail elderly Patient Outcomes on Dialysis (FEPOD) study did not find any differences between 129 asPD and 122 in-center HD matched patients during the 3 months running-in period before entering the study [30].

Data on hospitalization are then reported in different modalities and as absolute data making difficult to draw conclusions (Table 2).

**Table 2:** Outcomes of asPD

**Hospitalization**	
Country	Results
France [20]	31/36 have been hospitalized once during the study period (1998–2003)
	0.4 admission/patient/month
	46% have been hospitalized during the first 6 months. The percentage decreases to 21% after the first year
Denmark [22]	30,358 patients/day (64 asPD patients), 10% of which have been spent in hospital, with a length of stay longer for older (> 70 years) and for asPD compared with self-PD (35 vs 19 hospital days/patient/year)
	1 admission every 3.1 treatments/months
Brazil [25]	15/30 patients (50%) have been hospitalized during the first 6 months, with cardiovascular disease being the most common cause
Canada, Ontario [16]	1.4 admissions/patients/year and 23.5 hospital days/patient/year
Canada, Ontario [18]	203 asPD vs 198 in-center HD: 11.1 vs 12.9 days/year
Canada, British Columbia [19]	Non-significant differences between 53 asPD 57 PDA eligible* and 670 self-PD after adjusting for comorbidities
**Peritonitis risk**	
France [20]	Survival free of peritonitis was 72% at six months, 50% at 12 months
Denmark	Incidence of 1 in every 25.3 patients/months in asPD vs 1 in every 30 patients/months in self-PD patients
Canada, Ontario [16]	Non-significant differences between asPD and self-PD patients (1 in every 28.2 patients/months in self-PD vs 1 in every 24.9 patients/months)
Canada, British Columbia [19]	Non-significant differences between 53 asPD 57, PDA eligible* and 670 self-PD in both non-adjusted and adjusted analyses (0.22 vs 0.36 vs 0.22 patients-year)
Brazil [25]	1 episode in every 36 patients/months
**Technique survival**	
France [20]	85% at 6 months and 58% at 12 months
	8/36 were transferred to HD
Denmark [22]	No differences between self- and assisted PD patients
Canada, Ontario [16]	81% at 12 months
Canada, British Columbia [19]	Not-significant different between asPD, PDA eligible* and self-PD patients (88% vs 86% vs 83% of death- and transplant-censored PD technique survival at 12 months)
**Patient survival**	
France [20]	Patient survival was 90% at six months and 83%at 1 year
Denmark [22]	Patient survival was 75–80% at 1 year
Canada, Ontario [16]	Death rate 0.12 patients/year

asPD: assisted peritoneal dialysis; *PDA eligible: patients eligible for asPD but with service of assistance unavailable

One of the main concerns about asPD is the short training of assistants, which may increase the risk of peritonitis. However, it is described that peritonitis rate for asPD patients ranges between 1/25 and 1/36 patients months [22, 25, 31], which is well within the range of 1/18 patients months, recommended by ISPD guidelines [8]. Comparison between asPD and self-PD patients showed variable results, since in some reports peritonitis risk is increased in asPD, while in others assistance seems to be protective (Table 2).

Nephrologists usually underestimate patient-reported outcomes. Conversely, patients may give more importance to QoL than to survival, since dialysis has a great impact on daily life and may be experienced as a heavy burden, especially in older patients. A recent metanalysis including 4158 home-dialysis patients, mostly treated with PD, and 7854 in center HD patients, showed slight better QoL scores in home-dialysis patients compared with in-center HD patient, although a high heterogeneity across studies was found.

Quality of life and physical function has been specifically evaluated in 106 asPD patients older than 60 years, compared with 100 HD matched patients in FEPOD study [30]. They found no difference in QoL, but a higher treatment satisfaction in asPD compared HD patients.

Patients’ experience with asPD has also been evaluated through a semi-structured qualitative feedback in the program of British Columbia. They performed telephone interview to nine patients and their caregiver, asking about the assistance provided. Participants were all satisfied with the service and desired its continuation, giving value to the maintenance of independence and autonomy and to the relief from burden associated with self-PD.

Petersson and Lennerling analyzed life with asPD by patient’s perspective, conducing interview in 10 patients. Living with asPD compels the patient to face four major themes: facing new demands, managing daily life, partnership in care and experiencing a meaningful life. Despite being frail, elderly, physically dependent, their qualitative analysis showed that asPD patients “strive for maintaining well-being” and experience a good QoL. Both studies focused on the need of personalized model of care and continuity of nurses, as elements to be improved [32].

Finally, many studies make cost analysis of asPD, comparing it with in-center HD. Cost of assistance is extremely variable across the world depending on health care system organization, type and intensity of assistance and cost of life (Table 1). In France, where asPD is more expensive compared to other countries, the price of CAPD is around 23,400 Euros/year and 18,200 Euro/years for APD [33]. Despite the high cost, an analysis of Haute Autoritè de Santè demonstrated that cost of in-center HD is still higher than asPD, not even considering the capital cost [21]. Globally, asPD has been demonstrated to be cost-effective compared with in center HD. However, continuous and qualified assistance may increase cost of asPD, reaching the one of in-center HD. The Canadian program of asPD described by Oliver et al. reported an additional expense for assistance of 12,000 Canadian dollars (8260 Euros) for patient/year, with an average of 4.6 visits/week provided [16]. With two daily visits, the cost would triple, making asPD even more expensive than in center HD.

Few studies have then evaluated the effect of asPD on PD prevalence. In a Canadian experience, patients with access to a home-care assistance program were 2.6 time more likely to be considered eligible for PD, than patients from regions without home assistance [16, 34]. Provision of assistance have shown to increase PD utilization from 23 to 39% [34]. Boyer et al. analyzed PD utilization before and after asPD introduction in UK, finding that assistance service increased PD initiation by almost 80% (HR 1.78, CI 1.21–1.61). However, such rise was evident soon after service introduction, but flattened later on, showing that effect on PD prevalence may be less pronounced than expected [9].

## Conclusions

Assisted PD gives opportunities to patients with barriers to self-PD to choose home dialysis. It is mainly thought for elderly with barriers to self-PD, which may require assistance temporarily or on long-term basis. However, it is feasible for all patients who have lost independence and cannot perform alone PD, who otherwise would be temporarily shifted to HD.

Many strategies of asPD have been described, differing for type of assistants employed and intensity of assistance provided. Health care system organization, cost of life and type of assistance are responsible for differences in cost of asPD programs, which varies from 7280 euro/yearly in Canary Island (Spain) to 23,400 euro/yearly in France. It may be very expensive if full professional assistance is provided continuously, but if a mixed model of assistance focused on patients need is applied, significant costs saving may be obtained.

In an ideal mixed model of asPD, both health care and non-health care employers can be used, choosing the professional health care workers for more complex patients who need high level of assistance and less qualified workers for patients who need help only with physical tasks of PD. This system has been proven to be safe and cost-effective [16].

Clinical outcomes are difficult to be analyzed since data are reported in different ways and comparison are affected by bias. However, given the subset of patients, attention needs to be paid to their perception of well-being and to QoL considerations.

Even if given assistance may increase PD incidence, the effect of prevalence is low because asPD alone is ineffective in reverse PD decline which depends on many non-clinical reasons. The point is that we do not need asPD to increase PD utilization, but we need it to give patient the possibility to choose between more RRT alternative.

## Data Availability

Data sharing is not applicable to this article as no datasets were generated or analyzed during the current study.
